# Knowledge Distillation for Semantic Segmentation Using Channel and Spatial Correlations and Adaptive Cross Entropy

**DOI:** 10.3390/s20164616

**Published:** 2020-08-17

**Authors:** Sangyong Park, Yong Seok Heo

**Affiliations:** Department of Electrical and Computer Engineering, Ajou University, Suwon 16449, Korea; mailhoho@ajou.ac.kr

**Keywords:** semantic segmentation, knowledge distillation, channel and spatial correlation loss, adaptive cross entropy loss

## Abstract

In this paper, we propose an efficient knowledge distillation method to train light networks using heavy networks for semantic segmentation. Most semantic segmentation networks that exhibit good accuracy are based on computationally expensive networks. These networks are not suitable for mobile applications using vision sensors, because computational resources are limited in these environments. In this view, knowledge distillation, which transfers knowledge from heavy networks acting as teachers to light networks as students, is suitable methodology. Although previous knowledge distillation approaches have been proven to improve the performance of student networks, most methods have some limitations. First, they tend to use only the spatial correlation of feature maps and ignore the relational information of their channels. Second, they can transfer false knowledge when the results of the teacher networks are not perfect. To address these two problems, we propose two loss functions: a channel and spatial correlation (CSC) loss function and an adaptive cross entropy (ACE) loss function. The former computes the full relationship of both the channel and spatial information in the feature map, and the latter adaptively exploits one-hot encodings using the ground truth labels and the probability maps predicted by the teacher network. To evaluate our method, we conduct experiments on scene parsing datasets: Cityscapes and Camvid. Our method presents significantly better performance than previous methods.

## 1. Introduction

Semantic segmentation is a pixel-wise classification problem that determines a predefined class (or label) for each pixel in an image. This is a fundamental problem in the field of computer vision, and it can be applied to numerous real-world applications of vision sensors, including virtual reality, augmented reality, autonomous vehicles, aerial, and satellite image analysis.

Recently, numerous semantic segmentation methods that have exhibited reasonable performances are based on deep neural network algorithms. Since the seminal work of fully convolutional neural networks (FCNs) [1], numerous deep learning-based networks have been proposed for semantic segmentation [2,3,4,5,6,7,8,9,10,11,12,13,14]. In general, the deeper and wider the networks, the more accurate and improved the results. Thus, most of these methods focus on accuracy under all scenarios.

Moreover, with the success of deep learning-based methods, their applications in mobile environments have attracted significant interest. However, in mobile environments with embedded systems, there are inevitable limitations of hardware resources such as memory size and computational processing power compared to unrestricted general computers with large memory, multi-core CPU and high-performance GPUs. Thus, in these mobile environments, it is important to use less memory and have low computational complexity. Concurrently, the methods that generate accurate results generally require a large memory and heavy computations.

Hence, to satisfy the requirements of mobile environments, light but efficient deep-neural network-based methods have been proposed, including ENet [15], ICNet [16], ESPNet [17], ERFNet [18], and ESCNet [19]. The above methods can reduce the memory and the number of complexities while presenting accurate performances. Although these networks adopt computationally efficient methods, they have a limitation in that their accuracies are still lower than those of heavy networks. Furthermore, because these light networks are trained independently of the heavy networks, the knowledge in heavy networks cannot be transferred to light networks. To deal with this problem, one of the suitable strategies is knowledge distillation [20,21], which can assist in improving the accuracies of light student networks using the knowledge of heavy teacher networks. There are numerous knowledge distillation methods for classification tasks, and they have been verified to improve the network performance [20,22,23,24,25,26]. However, these methods are not appropriate to be directly applied to a semantic segmentation task, because the network structures of the above both tasks are inherently different. An image classification task aims to generate only one predefined label from a single image, whereas the objective of the semantic segmentation problem is to predict a label for each single pixel in the input image. Therefore, to predict dense results for all the pixels, the distillation methods for semantic segmentation networks are different compared to those for classification tasks. Consequently, several knowledge distillation methods have been proposed for semantic segmentation networks [27,28]. However, most of the previous methods have some limitations. First, they tend to transfer information of only the spatial relationship of the feature maps and ignore the channel relationship. The relation between a pair of channels for a feature map is also a significant information to transfer. Second, they are probable to transfer false knowledge and propagate error when the results of the teacher network are not perfect, because most of these methods directly transfer probability maps of the teacher network that might lead to inaccurate results.

In this paper, we propose a method to solve these two problems. First, to transfer the full relation of both the channel and spatial information in the feature map, we propose a channel and spatial correlation (CSC) loss function by computing channel and spatial correlation matrices. Second, we also propose an adaptive cross-entropy (ACE) loss function which adaptively exploits one-hot encodings using the ground-truth labels and probability maps based on the prediction of the teacher network.

**Figure 1:**
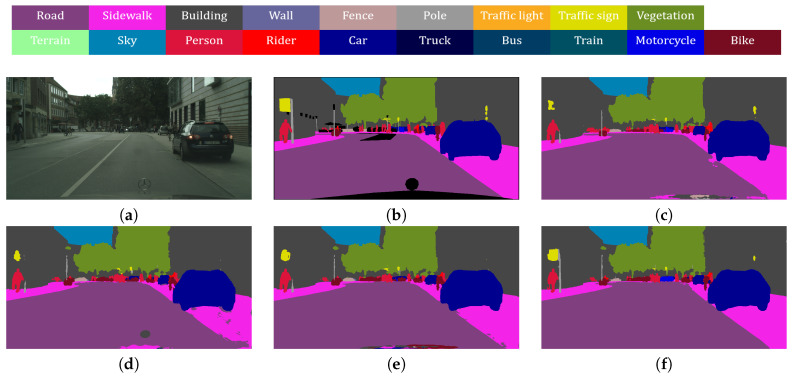
Comparison of the proposed distillation method and other methods. (**a**) Input image. (**b**) Ground truth. (**c**) Result of the teacher network. (**d**) Result of the student network without distillation. (**e**) Result of the student network using Pair-wise distillation [28]. (**f**) Result of the student network using the proposed distillation method.

Figure 1 illustrates the effects of our method. In this example, the teacher network is the heavy Deeplab-V3 + network [11] with Xception65 as the encoder, and the student network adopts the light Resnet34 as the encoder. The decoders of both the teacher and student networks are the same. The baseline is a student network that is trained by the conventional cross-entropy loss without the distillation method. Note that our method notably yields improved results. For regions where the teacher predicted incorrectly, our method can correct those regions. Also, for regions where previous distillation method [28] fails, our method generates more accurate results. Main contributions of this paper can be summarized as follows:

- We propose a channel and spatial loss function that transfers the full relation of both the channel and spatial information in the feature map from a teacher network to a student network.
- We propose an adaptive cross entropy loss function, which adaptively exploits the ground truth labels and prediction results of the teacher networks to prevent error propagation from it.

## 2. Related Work

In this section, we review the literatures that are related to our proposed method, including state-of-the-art methods for generic semantic segmentation, efficient semantic segmentation, and knowledge distillation.

### 2.1. Semantic Segmentation

Since the fully convolutional networks (FCNs) [1] were introduced, deep convolutional neural network (CNN)-based methods exhibited significantly improved performance for the semantic segmentation task. Most of the CNN-based methods for semantic segmentation consist of the contracting encoder and expanding decoder networks, where they are typically symmetric [2,4,29]. The encoder networks consist of repeated convolution and pooling layers for extracting the feature maps with reduced spatial resolution, whereas the decoder networks consist of multiple up-sampling layers to perform pixel-wise dense predictions. DeconvNet [2] proposed a method to learn a deconvolution network for preserving the detailed structures of the objects in an input image. U-Net [29] comprises of a U-shaped encoder–decoder network that combined symmetrical features originating from the corresponding encoder and decoder pair to perform precise dense prediction. SegNet [4] proposed a unpooling method that uses only the indices of the encoder using skip connections. These symmetric architectures have the same number of encoder and decoder layers. However, it is difficult to adopt encoders that are constructed using deep layers, such as Resnet101 [30] and Xception65 [31], because they require a significantly large memory. To employ deep and heavy encoders to semantic segmentation networks, most of the modern networks have adopted asymmetric architectures, which include a heavier encoder and a shallower decoder than those in symmetric architectures [10,11,12,32]. These asymmetric architectures have achieved higher accuracies and can optimize more rapidly using pre-trained weights for large datasets, such as Imagenet [33], than symmetric ones. Although the feature maps obtained from these deep encoder layers include a large amount of contextual information, the spatial resolution tends to be reduced. Thus, these networks have an inherently common problem in that the edge boundaries of the segment results are ambiguous. To deal with this, networks that combine both low- and high-level features have been proposed [8,9]. Ghiasi and Fowlkes [8] proposed a method to combine both low- and high-level features using a Laplacian pyramid and boundary masks. RefineNet [9] presented a residual convolutional unit, with a multi-resolution fusion and chained residual pooling for using multi-level features. However, some methods [10,11] focused on fusing feature maps that have various receptive fields. PSPNet [10] presented a pyramid pooling layer, which computes various receptive fields using multiple sizes of pooling layers. Deeplab-V3 + [11] proposed an atrous spatial pyramid pooling, which uses atrous convolution (or dilated convolution) to efficiently compute large and various receptive fields. Recently, some methods have improved the performance using an attention approach [12,32]. OCNet [12] employs an object context, which is defined as a set of pixels belonging to the same object category. The object context is adopted by object context pooling (OCP), which is added to a conventional pooling layers, such as pyramid pooling and atrous spatial pooling. DANet [32] involves a dual attention network that uses channel and spatial attention. The channel and spatial attention are computed by the relation of each channel and pixel, respectively. Generic semantic segmentation methods focus on performance of accuracy. Therefore, they require a lot of memory and a lot of computational complexity. In this study, through using these generic semantic segmentation knowledge, accuracy of efficient semantic segmentation networks improve.

### 2.2. Efficient Semantic Segmentation

Recently, as applications in mobile environments have become more important, numerous architectures that are more specialized in mobile environments for semantic segmentation have been proposed. To employ deep convolution neural networks in mobile environments, architectures must have reduced computational complexity and must use less memory. Therefore, it is difficult to apply heavy architecture that are designed for high accuracy in mobile environments.

To apply heavy architectures, such as PSPNet [10] and Deeplab-V3 + [11], in mobile environments, one of the approaches is to use a shallow encoder, such as Resnet18 [30], Mobilenet-V2 [34], and Sufflenet [35]. Alternatively, another approach is to construct mobile-specific architectures. These methods include efficient encoders and extremely shallow decoders compared to heavy architectures. Paszke et al. [15], inspired by [36], constructed an efficient network that includes a light weight encoder as the feature extractor and a small-sized decoder using a down-sampled input image. ICNet [16] proposed a method that divides the input images to low-, mid-, and high-resolution images using cascade feature fusion. Treml et al. [37] adopted the fire module proposed in [38] and parallel dilated convolution. ESPNet [17] employed efficient spatial pyramid of dilated convolutions for replacing general convolution layers. ContextNet [39] proposed a multi-branch network that fused the features of a deep network at a small resolution and those of a shallow network at full resolution. These methods have reduced the computational complexity and increased the running speed.

Concurrently, to satisfy rich spatial information and a sizeable receptive field, BiseNet [40] comprises an architecture that has two paths: context and spatial path. The context path provides sufficient receptive fields, while the spatial path preserves the spatial information in the original input image. Fast-SCNN [41] employs a pseudo two-branch architecture using the skip connection. It consists of a learning to down-sample module, which is a coarse global feature extractor, feature fusion module, and standard classifier. ESCNet [19] utilizes an efficient spatio-channel dilated convolution (ESC) module, which is an efficient multi-level dilated convolution module, to accomplish various receptive fields with reduced network parameters and computational complexity. EFSNet [42] propose the continuous shuffle dilated convolution (CSDC) module for less calculational effort. Efficient semantic segmentation methods focus on speed or low usage of memories. Therefore, generally, they have lower accuracy than generic semantic segmentation methods. In our study, we propose the knowledge distillation method to improve an accuracy of efficient methods using the knowledge of generic methods, which have better accuracy.

### 2.3. Knowledge Distillation

Knowledge distillation is a method that transfers knowledge of a cumbersome teacher network to a compact student network for rapid optimization and performance improvement of the student network. It was introduced by [20], which proposed a method to create a probability map of the teacher network and compared it to a probability map of the student network. Since then, numerous knowledge distillation methods have been proposed. Romero et al. [22] presented a hint-based learning method called FitNet, which compares the feature maps obtained from intermediate hidden layers of the teacher network with those of the student network. Zagoruyko and Komodakis [26] proposed a method to transfer knowledge by creating an one-channel attention map that uses an intermediate layer of the teacher and student. With the attention map, they do not require convolutions in the knowledge distillation term and achieve better performance. Yim et al. [23] proposed to help initialize a student network using the Gramian matrix between the layers to transfer the relation of layers. Wang et al. [25] proposed KDGAN, which applied generative adversarial networks (GAN) in the knowledge distillation method. However, most of these knowledge distillation methods have been applied to classification tasks.

Recently, a few methods have adopted knowledge distillation for the semantic segmentation problem. Xie et al. [27] proposed zero- and first-order knowledge. Zero-order knowledge is a method that induces the class probabilities for each pixel separately to transfer the knowledge. Concurrently, the first-order knowledge computes the difference between the neighboring pixels and transfer those information. Liu et al. [28] proposed pair-wise and holistic distillation schemes to enforce pair-wise and higher-order spatial consistency between outputs of the teacher and student networks, respectively. Knowledge distillation methods focus on classification problem. Some methods are adopted in semantic segmentation, but they ignore the relation of each channel and they transfer the error of the teacher network to the student network. In this study, we propose new knowledge distillation methods, which use channel and spatial correlation and adaptively cross entropy.

## 3. Our Approach

**Figure 2:**
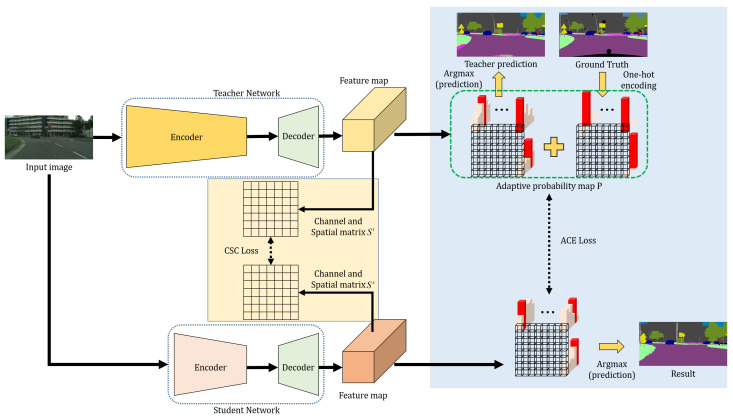
Framework of our proposed knowledge distillation method. Both the structures of the teacher and the student network have the same architecture, as Deeplab-V3 + [11], but different encoders. The depth of the encoder of the student network is shallower than that of the teacher network. Using the proposed CSC loss function, we can efficiently transfer the spatio-channel information of the teacher network to the student network. Using the proposed ACE loss function, the probability map result of the teacher network and ground-truth values can be adaptively transferred to the student network.

In this section, we describe our proposed method, which can maintain the speed and the memory of the light student network while improving its accuracy using the knowledge of the heavy teacher network. An overview of our method is depicted in Figure 2. We set the teacher network to have a deeper encoder than the student network, whereas both networks have the same decoder. To transfer the knowledge of the teacher network to the student network, we compute the channel and spatial correlation matrices. With these matrices, we propose a CSC loss function. In addition, to adaptively transfer the one-hot encodings using ground truth labels and the final probability maps of the teacher network, an ACE loss function is proposed. The proposed loss functions are explained in detail in the following subsections.

### 3.1. Channel and Spatial Correlation Loss Function

Let us denote a feature map of a final layer in a decoder as $z\in R^{W\times H\times C}$, where *W* and *H* are the width and height of the feature map, respectively, and *C* is the number of channels. To transfer the knowledge which maintains the channel and spatial relationship of feature maps from the teacher network to the student network, we compute channel and spatial correlation for the feature map.

**Figure 3:**
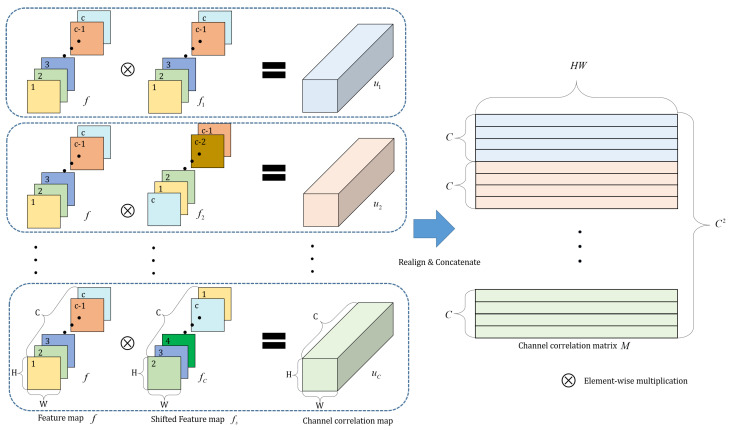
Illustration of the method to form the channel correlation matrix *M*. The feature map *f* is the normalized logit of network. The shifted feature map $f_{s}$ is obtained by shifting *f* along the channel axis. The channel correlation map *u* is obtained by multiplying the feature map *f* and the shifted feature map $f_{s}$. All the channel correlation maps $u_{s}$ are concatenated along the channel axis to form a total channel correlation map *u*, which is converted to the channel correlation matrix *M* by rearranging it.

First, each vector $z\left(i,j\right)\in R^{C}$ for each spatial position $\left(i,j\right)\in R^{W\times H}$ in *z* is normalized along the channel dimension to obtain a normalized feature vector $f\left(i,j\right)\in R^{C}$ as follows:

(1) $$f\left(i,j\right)=\frac{z(i,j)}{{∥z(i,j)∥}_{2}},$$

where ${∥\cdot ∥}_{2}$ is the L2-norm operation. To obtain the channel relationship information for each spatial position (i,j) in the feature map, we define a channel correlation vector, $u_{s}\left(i,j\right)\in R^{C}$ as follows:

(2) $$u_{s}\left(i,j\right)=f\left(i,j\right)⊗f_{s}\left(i,j\right),$$

where $f_{s}\left(i,j\right)$ is a circularly shifted vector with a shift of s−1 from the original vector f(i,j), as depicted in Figure 3, and ⊗ represents an element-wise multiplication operation. By concatenating $u_{s}\left(i,j\right)$ with *C* different numbers of *s*, we can obtain the total channel correlation vector $u\left(i,j\right)\in R^{C^{2}}$ as follows:

(3) $$u\left(i,j\right)=u_{1}\left(i,j\right)⊕u_{2}\left(i,j\right)⊕\cdots ⊕u_{C}\left(i,j\right),$$

where ⊕ denotes the concatenation operation.

Subsequently, by rearranging the total channel correlation vectors in all the spatial positions, the two-dimensional (2D) channel correlation matrix $M\in R^{WH\times C^{2}}$ can be obtained as displayed in Figure 3. Using *M*, we can construct a 2D channel and spatial correlation matrix $S\in R^{WH\times WH}$ as follows:

(4) $$S=MM^{T}.$$

Thus, the proposed CSC loss function $\ell_{CSC}$ is defined by

(5) $$\ell_{CSC}=\frac{1}{{\left(WH\right)}^{2}}\sum_{y=1}^{WH}\sum_{x=1}^{WH}{∥s^{t}\left(x,y\right)-s^{s}\left(x,y\right)∥}_{2}^{2},$$

where $s^{t}\left(x,y\right)$ and $s^{s}\left(x,y\right)$ are the ${\left(x,y\right)}^{th}$ element values of the channel and spatial correlation matrix $S^{t}$ and $S^{s}$ of the teacher network and the student network, respectively.

### 3.2. ACE Loss Function

**Figure 4:**
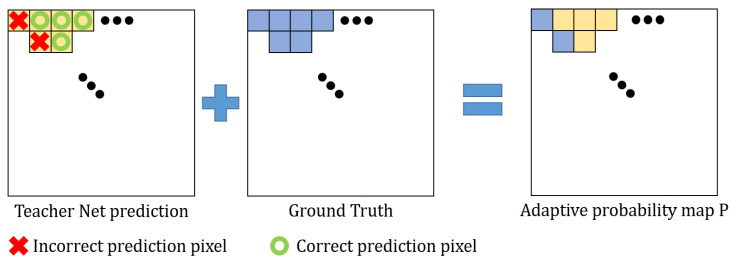
Illustration of generating an adaptive probability map. To generate the adaptive probability map, we check the teacher network prediction result as if it is a correct pixel. We use this new probability map for the proposed adaptive cross entropy (ACE) loss function.

Generally, the inference results of the teacher networks are not always accurate for every pixel. In this case, false knowledge of the teacher networks can be transferred to the student networks. Inspired by this observation, we propose an adaptive probability map *P* based on the prediction of the teacher network. As shown in Figure 4, for correctly predicted pixels of the teacher network, we use a weighted average of the probability vectors of the teacher network and one-hot encoded vectors using ground truth labels, which encourages more effective training [20]. Meanwhile, for incorrectly predicted pixels of the teacher network, we only use the one-hot encoded vectors using ground truth labels to block transferring error of the teacher network.

Thus, our adaptive probability vector $P\left(i,j\right)\in R^{C}$ for the ${\left(i,j\right)}^{th}$ pixel constituting $P\in R^{W\times H\times C}$ is defined by

(6) $$P\left(i,j\right)=\left\{\begin{array}{c} \kappa \cdot p^{t}\left(i,j\right)\;+\left(1-\kappa \right)\cdot p^{g}\left(i,j\right)\;\;\;\;\;\;if\;L^{t}\left(i,j\right)=G\left(i,j\right)\\ p^{g}\left(i,j\right)\;\;\;\;\;\;\;\;\;\;\;\;\;\;\;\;\;\;\;\;\;\;\;\;\;\;\;\;\;\;\;\;\;\;\;\;\;\;\;\;\;\;\;\;otherwise \end{array}\right.,$$

where $p^{t}\left(i,j\right)\in R^{C}$ is the probability vector computed using the softmax operation of the feature map of the final layer in the teacher network, and $p^{g}\left(i,j\right)\in R^{C}$ is the one-hot encoded vector using the ground truth label for the ${\left(i,j\right)}^{th}$ pixel, respectively. And, κ controls the weight between $p^{t}\left(i,j\right)$ and $p^{g}\left(i,j\right)$. G(i,j) is the ground truth label for the ${\left(i,j\right)}^{th}$ pixel, and $L^{t}\left(i,j\right)$ represents the predicted label of the teacher network that is defined by

(7) $$L^{t}\left(i,j\right)=\underset{c\in C}{\arg\max}p_{c}^{t}\left(i,j\right),$$

where $p_{c}^{t}\left(i,j\right)$ is the $c^{th}$ channel value of $p^{t}\left(i,j\right)$.

Using the adaptive probability map *P*, our ACE loss function $\ell_{ACE}\left(i,j\right)$ for the ${\left(i,j\right)}^{th}$ pixel is defined by

(8) $$\begin{array}{c} \ell_{ACE}\left(i,j\right)=-\sum_{c\in C}\left(P_{c}\left(i,j\right)\log p_{c}^{s}\left(i,j\right)\right), \end{array}$$

where $p_{c}^{s}\left(i,j\right)$ represents the $c^{th}$ channel value of $p^{s}\left(i,j\right)\in R^{C}$ which is the probability vector of the student network defined similar to $p^{t}\left(i,j\right)$. $P_{c}\left(i,j\right)$ represents the $c^{th}$ channel value of P(i,j) defined in Equation (6).

Thus, our ACE loss function $\ell_{ACE}$ is defined by

(9) $$\ell_{ACE}=\frac{1}{WH}\sum_{j=1}^{H}\sum_{i=1}^{W}\ell_{ACE}\left(i,j\right).$$

### 3.3. Total loss function

Now, the total loss function $\ell_{all}$ in our method is defined by

(10) $$\ell_{all}=\lambda_{CSC}\cdot \ell_{CSC}+\lambda_{ACE}\cdot \ell_{ACE},$$

where $\lambda_{CSC}$ and $\lambda_{ACE}$ are weighting factors of $\ell_{CSC}$ and $\ell_{ACE}$, respectively.

## 4. Experiments

We exploited Deeplab-V3+ [11] structure as the teacher network, because it is one of the state-of-art networks in the semantic segmentation task. The encoder of this network is Xception65 [31], which is deep and requires heavy computation. As is the case with recent semantic segmentation networks, the decoder of this network is relatively shallower than the encoder, and it consists of the atrous spatial pyramid pooling layer and up-sampling module. For the encoders of the student networks, we used shallow and light networks, including Resnet18, Resnet34 [30], and Mobilenet-V2 [34] to demonstrate the effectiveness of our distillation method. We fixed the decoders of these student networks to be the same as that of the teacher network.

### 4.1. Dataset

The Cityscapes [43], and Camvid [44] datasets are the standard datasets for the semantic segmentation task, and they are used to evaluate and compare the performance of our method with those of other methods. The Cityscapes dataset contains street images of urban scenes, which are exploited in most of the networks for semantic segmentation tasks. The labels of the dataset are composed of 30 classes, and only 19 of them are used for the scene parsing evaluation. The dataset contains 5000 high-quality images with pixel-level fine annotation and 20,000 coarsely annotated images. In this study, we exploit finely annotated images, which are divided into 2975 training, 500 validation, and 1525 test images. The Camvid data also contains the urban scenes for vehicle, and it comprises 32 classes, and only 12 of them are used for the scene parsing evaluation. The dataset contains 367 training, 101 validation, and 233 test images.

### 4.2. Training Setup

For reasonable comparisons of our method and other methods, we fixed hyper parameters such as the learning rate, mini-batch size, cropping size, and number of epochs, except for the structures of the encoder networks of the students. For training the student networks, we used four titan-x GPUs for the experiments.

For the Cityscapes dataset, the student networks were trained by the stochastic gradient descent (SGD) with the momentum of 0.9 and weight decay of 0.00005 for 250 epochs with mini-batch size 12. Here, we employed the poly learning rate policy [5,6], and the learning rate for training was initialized as 0.007. The new learning rate was computed by $lr_{new}=lr_{currnt}*{\left(1-\frac{iter}{total\_iter}\right)}^{0.9}$. When training the student networks, we used a random scaling factor ranging from 0.5 to 2.0, and cropped 709 × 709 size from the input images. We empirically set the weighting factor κ in Equation (6) as 0.5, and $\lambda_{CSC}$ and $\lambda_{ACE}$ in Equation (10) as 5 and 1, respectively.

Similarly, for the Camvid dataset, most of the training parameters are the same as those for the Cityscapes dataset, except the batch size, start learning rate, and cropped size. In the Camvid dataset experiments, we used a mini-batch size of 16, start learning rate of 0.005, and cropping size of 512 × 512.

### 4.3. Evaluation Metrics

To evaluate and compare the performances of various methods, we measured the segmentation accuracy, model size, and complexity of the network parameters. For accuracy, the intersection over union (IoU) score was used. It is defined by the ratio of the interval and union between the ground truth mask and the predicted segmentation mask for each class. This score is adopted by all of the semantic segmentation networks. The mean IoU (mIoU) calculates the average of all the classes IoU over all the images. We also compare each class IoU score to study the effects of our method on different classes. Concurrently, the model size is represented by the number of network parameters, and the computational complexity is evaluated with the sum of the floating point operations (FLOPs) in one forward pass on a 512 × 1024 cropped image on the Cityscapes dataset.

### 4.4. Ablation Study

#### 4.4.1. Effects of Each Loss Function

To investigate the effects of our loss function, we performed various ablation studies by enabling different terms in Equation (10). To this end, we tested with the Cityscapes dataset, and fixed the encoder architecture of the teacher and the student network, as Xception65 and Resnet34, respectively. mIoUs of validation, training, and test images for the teacher and the student networks using various loss functions are displayed in Table 1. “Resnet34 (CE)” represents the result of the student network with Resnet34 as the encoder using the conventional cross-entropy (CE) loss function without the knowledge distillation. “Resnet34 (CSC + CE)” signifies the result of the student network using the proposed CSC loss function in Equation (5) and the conventional CE loss function with ground truth labels, instead of ACE loss function in Equation (9). “Resnet34 (ACE)” represents the result of the student network using only ACE loss function. Concurrently, “Resnet34 (CSC + ACE)” represents the result of our method using both the CSC and ACE loss functions, as defined in Equation (10).

Table 1 exhibits that our distillation loss function significantly improves the performance of the student network. By comparing “Resnet34 (CE)” and “Resnet34 (CSC + CE)”, it can be noted that our CSC loss function helps in improving the mIoU of the student network by 1.05%, 0.44%, and 1.56% on validation, training, and test images, respectively. Meanwhile, by comparing “Resnet34 (CE)” and “Resnet34 (ACE)”, it was observed that our “ACE only” loss helps increase mIoUs of the student network than those of “CE only” by 4.9%, 3.99%, and 6.35% on validation, training, and test images, respectively. Thus, it is clear that ACE loss function contributes more than CSC loss function for improving accuracies of the student networks. However, by comparing the “ACE only”, “CSC + ACE”, and “CSC + CE” loss functions, “CSC + ACE” shows the best improvement of mIoU of the student network. Therefore, our CSC loss function is also necessary to obtain the best results.

**Figure 5:**
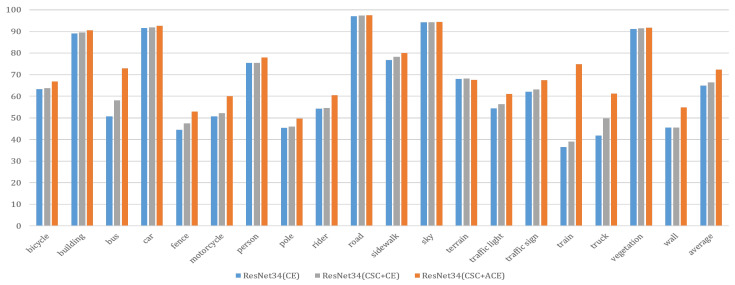
Illustration of the improvement of the accuracy using our distillation method for each class on the Cityscapes test set. We used the Deeplab-V3 + structure with Resnet34 as the encoder for this experiment. The blue bar (baseline) indicates the results without distillation method. The gray bar represents the results using the CSC + CE loss function. The orange bar represents results using the CSC + ACE loss function. The height of the bars represents mIoU(%).

Figure 5 displays that our method (“Resnet34 (CSC + ACE)”) clearly improves performance of every class compared with the baseline method (“Resnet34 (CE)”). In particular, several classes including bus, motorcycle, train, truck and wall are improved significantly. Note that the images with these classes consist of numerous textureless and confusing regions where large receptive fields are required to generate the accurate results. In these problematic regions, it can be seen that our method helps distinguish these confusing classes and effectively improves the accuracy compared to the baseline method.

**Figure 6:**
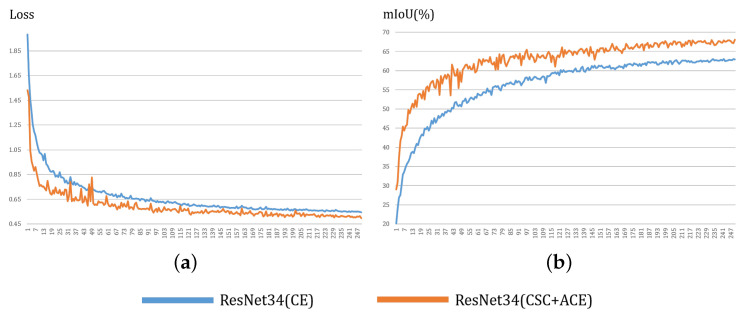
Effects of our distillation method on the speed of the optimization of the loss and accuracy for the Cityscapes validation set. These results were obtained for 709 × 709 cropped input images. (**a**) is cross-entropy loss graph per epoch and (**b**) is mIoU per epoch. The orange line is the result obtained using our distillation method, and the blue line (baseline) is without distillation method. Here, we adopt the Deeplab-V3 + structure, where Resnet34 is used as an encoder. The speed of the optimization of the loss and mIoU are significantly increased using our distillation method than those of the baseline method.

Apart from accuracy, our method (“Resnet34 (CSC + ACE)”) also facilitates the faster optimization of the student networks. In Figure 6a, for a fair comparison of each method, we used the same loss values as the conventional CE loss using the logits in the final layers from the predicted results. Figure 6 displays that the loss values of our method decrease faster than the baseline method (“Resnet34 (CE)”), whereas mIoU of our method increases rapidly than that of the baseline method.

#### 4.4.2. Effects of the Number of Channels of the Feature Map

To investigate the effects of the channel number of the feature map on our CSC loss function, we performed two experiments. First, we eliminated the repeated elements in the total channel correlation vector $u\left(i,j\right)\in R^{C^{2}}$ in Equation (3) and generated a new vector $v\left(i,j\right)\in R^{\frac{C(C+1)}{2}}$ from u(i,j) for each ${\left(i,j\right)}^{th}$ position in the feature map. Note that there are duplicated elements in the vector u(i,j), because the vector u(i,j) is a 1D vector where each element constitutes the C×C gram matrix *U* generated from a vector $f\left(i,j\right)\in R^{C}$ in Equation (1). The gram matrix *U* is a symmetric matrix where upper triangular and lower triangular elements are duplicated except the diagonal elements. Thus, we eliminated either of the duplicated elements from u(i,j) to generate v(i,j) for both the teacher and the student networks. Consequently, we define a new CSC loss function “CSC_Eli” using v(i,j) instead of u(i,j) that is similarly defined as Equation (5). In Table 2, “Resnet34 (CSC_Eli + CE)” represents the results using “CSC_Eli” and CE. From this experiment, it was observed that our original CSC method (“Resnet34 (CSC + CE)”) using u(i,j) generates higher mIoU results than “Resnet34 (CSC_Eli + CE)” using v(i,j) by 1.27%, 0.66%, and 1.63% on validation, training, and test images on Cityscapes dataset, respectively. It is worth to note that all the repeated elements in u(i,j) corresponds to the off-diagonal elements in the gram matrix, and they include relationships between two different channels in the feature map. Therefore, the repeated elements in the channel correlation vector u(i,j) allows our CSC function to further emphasize the relationships between different channels and help increase accuracy.

Second, we reduced the channel size of the feature vector $f\left(i,j\right)\in R^{C}$ generated by decoders of the teacher and the student networks using pooling operation for each ${\left(i,j\right)}^{th}$ position in the feature map. Specifically, to construct the compact feature vector $\hat{f}\left(i,j\right)\in R^{\frac{C}{2}}$ with half the channel size, we performed max pooling operation with kernel size of 2 only on the channel axis from the feature vector f(i,j). From Table 2, “Resnet34 (CSC_Pooling + CE)” represents results of the CSC method using the compact feature vector $\hat{f}\left(i,j\right)$. The results using our original CSC method “Resnet34 (CSC + CE)” generates slightly better mIoUs than “Resnet34 (CSC_Pooling + CE)” by 0.49% and 0.04% on validation and test images on Cityscapes dataset, respectively. Although the channel information is reduced in the feature vector $\hat{f}\left(i,j\right)$, important information to determine class (or label) is still preserved even after the max pooling operation. Thus, the mIoUs of “Resnet34 (CSC_Pooling + CE)” are slightly worse than those of “Resnet34 (CSC + CE)”.

#### 4.4.3. Effects of Architectures of Student Networks

To investigate the effects of our loss function on the student networks, we performed various experiments. Table 3 shows the performances of current state-of-the-art networks for semantic segmentation without knowledge distillation. In Table 3, it was observed that the light networks require fewer parameters and FLOPs, but are less accurate than the heavy networks. Table 4 compares the accuracy of each student network with and without our distillation method in terms of mIoUs for validation, training, and test images on the Cityscapes dataset, respectively. In Table 4, further measurements are also provided including the number of parameters, FLOPs, processing time, and memory usage.

“Resnet34” represents a student network with an encoder of Resnet34 using the conventional CE loss without the knowledge distillation. Concurrently, “Resnet34 (ours)” refers to the student network with an encoder of Resnet34 using our knowledge distillation loss function in Equation (10). For the other networks such as Resnet18 and Mobilenet-V2, similar notations are used.

The FLOPs are calculated at a resolution of 512 × 1024 size of image to evaluate the computational complexity, and #parameters is the number of network parameters for measuring the size of the network. The processing time and memory usage represent inference time and consumed memory of each network for a single image with size of 512 × 1024, respectively. Because our method does not change the network architecture of the student, FLOPs, number of parameters, processing time, and memory usage are the same between the methods with and without our distillation loss function.

It is worth noting that our method significantly improves the accuracies of the student networks compared to the baseline method which is optimized using the conventional CE loss without the knowledge distillation. When we adopt our method in the Resnet34 student network, it improves 5.34%, 4.70%, and 7.49% of mIoU compared to the method without knowledge distillation on the validation, training, and test images, respectively. For the Resnet18 network, the mIoUs of the validation, training, and test images increase by 5.81%, 6.83%, and 6.60% compared to the baseline method. Similarly, for the Mobilenet-V2 network, the mIoUs of validation, training, and test images increase by 7.70%, 5.61%, and 7.28%, respectively, compared to the baseline method.

We also experimented on the Camvid dataset to demonstrate the effectiveness of our distillation method. From Table 5, we can see that the results of our method improve performance significantly. When we adopt our method in the Resnet34 student network, it improves 7.22%, 11.11%, and 7.35% of mIoU compared to the method without distillation on the validation, training, and test images, respectively. For Resnet18 network, the mIoUs of validation, training, and test images increase by 9.81%, 11.88%, and 7.97%, respectively. For Mobilenet-V2 network, the mIoUs of validation, training, and test images increase by 10.33%, 13.54%, and 8.88%, respectively, compared to the baseline method.

From these experiments, it can be noted that the improvements of the accuracies using our method are clearer for networks with small sizes of parameters.

### 4.5. Comparative Results

To evaluate the performance of our method, we compared our method with other distillation methods. For reasonable comparisons, the networks of the student were fixed identically, and only the loss functions were different. Here, we used Resnet34 as the encoder of the student network. To determine the effects of our CSC loss function in Equation (5), we replace our CSC distillation term with other distillation schemes including [22,28]. In addition, the effects of the proposed ACE loss function were evaluated by replacing it with the conventional CE loss function. Table 6 and Table 7 summarize the comparison results of the student network obtained by varying distillation loss functions on the Cityscapes and Camvid datasets, respectively. In Table 6 and Table 7, “CE” represents a method using only the CE loss function using ground-truth labels without knowledge distillation. “MIMIC [22] + CE” is a method of feature distillation by MIMIC [22] combined with CE. When we performed feature distillation using MIMIC, we normalized the logits of the features of the teacher and student, and computed the L2 distance between them. Similarly, “Pair-wise [28] + CE” is a method of the global pair-wise feature distillation [28] combined with CE. The “Pair-wise” distillation transfers all the pair-wise spatial dependencies which are computed by spatial correlation matrix in the feature map. Thus, it is calculated by L2 distance between corresponding elements in the spatial correlation matrices of the teacher and the student.

In the MIMIC [22] method, there are not any information of relationship (or correlation) between features. The “Pair-wise” [28] method includes relationships between features only in the spatial dimension. These constraints on the spatial relationship can achieve semantic consistency between pixels. However, this distillation loss does not consider relation of channels unlike our CSC distillation function. Meanwhile, our CSC loss can capture more discriminative properties of the features by capturing interdependencies of spatial domain as well as channel domain. Because the channels of the features in the decoder includes class-specific responses [32], the interdependency between channels additionally increase semantic discriminability.

From Table 6 and Table 7, it can be seen that our distillation method generates more accurate results than other methods. By comparing “CSC + CE” and other feature distillation schemes with CE, we can see that our CSC loss function is slightly better than other methods. Our CSC loss function efficiently captures contextual information in both channel and spatial domains rather than only spatial domain. Meanwhile, performances of all the feature distillation methods combined with the proposed ACE loss are significantly improved compared to the methods with the conventional CE. These improvements of accuracy are mainly from the adaptive property of our ACE loss function which does not suffer from the errors of the teacher network.

**Figure 7:**
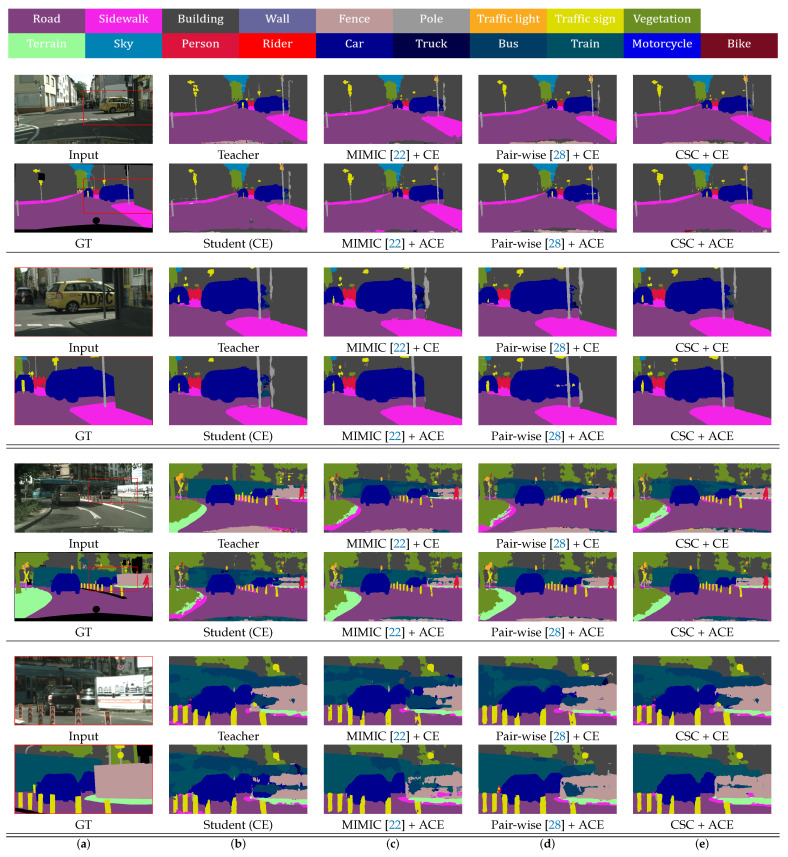
Qualitative comparison of various methods on the Cityscapes validation set. (**a**) Input and ground-truth (GT) images. (**b**) Teacher and student (CE) networks. (**c**) MIMIC [22]. (**d**) Pairwise [28]. (**e**) Proposed method.

**Figure 8:**
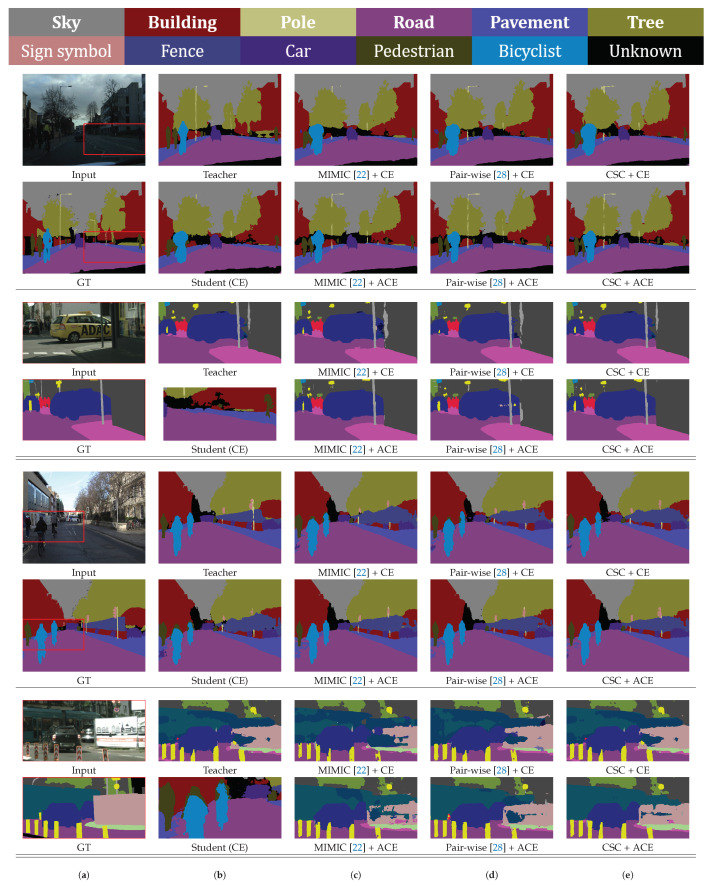
Qualitative comparison of various methods on the Camvid validation and test set. (**a**) Input and ground-truth(GT) images. (**b**) Teacher and student (CE) networks. (**c**) MIMIC [22]. (**d**) Pair-wise [28]. (**e**) Proposed method.

From a qualitative point of view, Figure 7 and Figure 8 demonstrate some of comparative results, where results of the original input images and corresponding magnified images for the red rectangle regions in the input images are shown on the Cityscapes and Camvid datasets, respectively. Note that the results of our method are less noisy, and the edges of the results are more distinct than those of the other methods. Our CSC loss function helps to obtain more accurate results than other feature distillation methods for ambiguous and confusing regions such as road, sidewalk, and pavement. In addition, for regions where the teacher network fails such as pole, fence, and traffic sign, our ACE loss function efficiently prevents errors of the teacher network from propagating to the student networks, and corrects them. This obviously improves accuracies of student networks compared to using the CE loss function.

## 5. Conclusions

In this paper, we explore knowledge distillation for training compact semantic segmentation networks using heavy networks. We present two distillation loss functions: channel and spatial correlation (CSC) distillation loss and adaptive cross-entropy (ACE) distillation loss. Our CSC loss function can capture more discriminative properties of the features by encoding dependencies of both spatial and channel domains. In addition, our ACE loss function improves the accuracy of the student network by adaptively exploiting the ground truth labels and the probability maps predicted by the teacher network. Various experiments demonstrate the effectiveness of our proposed distillation loss functions for compact student networks on the Cityscapes and Camvid datasets. Specifically, we demonstrate that our CSC loss function helps increase the accuracy of the student network for ambiguous and confusing regions compared to previous methods. In addition, our ACE loss function significantly increases the accuracy of the student network by effectively preventing the errors of the teacher network.

## Figures and Tables

**Table 1:** Effects of different components of the loss in the proposed loss function. CSC is the channel and spatial correlation loss, ACE is the adaptive cross-entropy loss, and CE is the conventional cross-entropy loss. Our experiments were initialized from pretrained weights on ImageNet.

Method	Val. mIoU (%)	Train. mIoU (%)	Test. mIoU (%)
Teacher	74.43	79.23	72.55
Resnet34 (CE)	67.94	72.35	64.87
Resnet34 (CSC + CE)	68.99	72.79	66.43
Resnet34 (ACE)	72.88	76.34	71.22
Resnet34 (CSC + ACE)	73.28	77.05	72.36

**Table 2:** Effects of the number of channels in the feature map.

Method	Val. mIoU (%)	Train. mIoU (%)	Test. mIoU (%)
Teacher	74.43	79.23	72.55
Resnet34 (CSC + CE)	68.99	72.79	66.43
Resnet34 (CSC_Eli + CE)	67.72	72.13	64.80
Resnet34 (CSC_Pooling + CE)	68.50	72.88	66.39

**Table 3:** Results of numerous current state-of-the-art networks.

Network	#Params (M)	FLOPs (G)	Val. mIoU (%)	Train. mIoU (%)	Test. mIoU (%)
ERFNet [18]	2.067	30.18	71.5	n/a	68.0
ICNet [16]	28.30	74.02	67.7	n/a	69.5
ESPNet [17]	0.36	5.55	61.4	n/a	60.3
BiseNet [40]	5.8	30.35	74.8	n/a	74.7
Fast-SCNN [41]	1.11	1.91	69.22	n/a	68.0
SegNet [4]	29.45	326.77	n/a	n/a	56.1
PSPNet [10]	49.08	369.49	78.38	n/a	78.4
DANet [32]	68.50	552.67	81.50	n/a	81.5
OCNet [12]	62.54	613.15	79.58	n/a	80.1

**Table 4:** Effects of our distillation method for various student networks on the Cityscapes dataset.

Network	#Params (M)	FLOPs (G)	Val. mIoU (%)	Train. mIoU (%)	Test. mIoU (%)	Proc. Time (s)	Memory Usage (GB)
Teacher	41.05	104.03	74.43	79.23	72.55	0.1116	8.19
Resnet34	22.45	69.30	67.94	72.35	64.87	0.0382	2.09
Resnet34 (ours)			73.28	77.05	72.36		
Resnet18	12.34	42.66	64.84	69.66	63.10	0.0299	1.81
Resnet18 (ours)			70.65	76.49	69.70		
Mobilenet-V2	2.25	15.85	58.60	62.59	57.43	0.0292	2.41
Mobilenet-V2 (ours)			66.30	68.20	64.71		

**Table 5:** Effects of our distillation method on various student networks on the Camvid dataset.

Network	Val. mIoU (%)	Train. mIoU (%)	Test. mIoU (%)
Teacher	75.05	81.11	70.73
Resnet34	62.96	65.88	57.90
Resnet34 (ours)	70.18	76.99	65.25
Resnet18	58.59	63.19	55.63
Resnet18 (ours)	68.40	75.07	63.60
Mobilenet-V2	58.10	59.83	51.79
Mobilenet-V2 (ours)	68.43	73.37	60.67

**Table 6:** Comparison of different distillation methods on the Cityscapes dataset. CE is the method that uses the conventional cross-entropy loss function without distillation. CSC is the method using the proposed CSC loss function. ACE is the method using the proposed ACE loss function.

Method	Val. mIoU (%)	Train. mIoU (%)	Test. mIoU (%)
CE	67.94	72.35	64.87
MIMIC [22] + CE	68.59	72.37	65.31
Pair-wise [28] + CE	68.90	72.58	66.03
CSC + CE	68.99	72.79	66.43
MIMIC [22] + ACE	73.04	76.84	71.75
Pair-wise [28] + ACE	73.25	77.00	72.25
CSC + ACE	73.28	77.05	72.36

**Table 7:** Comparison of different distillation methods on the Camvid dataset. CE is the method using the conventional cross-entropy loss function without distillation. CSC is the method using the proposed CSC loss function. ACE is the method using the proposed ACE loss function.

Method	Val. mIoU (%)	Train. mIoU (%)	Test. mIoU (%)
Teacher network	75.05	81.11	70.73
CE	62.96	65.88	57.90
MIMIC [22] + CE	64.17	67.38	59.67
Pair-wise [28] + CE	65.12	68.36	59.83
CSC + CE	65.53	68.47	60.40
MIMIC [22] + ACE	69.89	76.73	65.00
Pair-wise [28] + ACE	69.32	77.20	65.02
CSC + ACE	70.18	76.99	65.25
